# The Cell Cycle Independence of HIV Infections Is Not Determined by Known Karyophilic Viral Elements

**DOI:** 10.1371/journal.ppat.0010018

**Published:** 2005-11-11

**Authors:** Masahiro Yamashita, Michael Emerman

**Affiliations:** Division of Human Biology and Basic Sciences, Fred Hutchinson Cancer Research Center, Seattle, Washington, United States of America; King′s College London, United Kingdom

## Abstract

Human immunodeficiency virus and other lentiviruses infect cells independent of cell cycle progression, but gammaretroviruses, such as the murine leukemia virus (MLV) require passage of cells through mitosis. This property is thought to be important for the ability of HIV to infect resting CD4+ T cells and terminally differentiated macrophages. Multiple and independent redundant nuclear localization signals encoded by HIV have been hypothesized to facilitate migration of viral genomes into the nucleus. The integrase (IN) protein of HIV is one of the HIV elements that targets to the nucleus; however, its role in nuclear entry of virus genomes has been difficult to describe because mutations in IN are pleiotropic. To investigate the importance of the HIV IN protein for infection of non-dividing cells, and to investigate whether or not IN was redundant with other viral signals for cell cycle-independent nuclear entry, we constructed an HIV-based chimeric virus in which the entire IN protein of HIV was replaced by that of MLV. This chimeric virus with a heterologous IN was infectious at a low level, and was able to integrate in an IN-dependent manner. Furthermore, this virus infected non-dividing cells as well as it infected dividing cells. Moreover, we used the chimeric HIV with MLV IN to further eliminate all of the other described nuclear localization signals from an HIV genome—matrix, IN, Viral Protein R, and the central polypurine tract—and show that no combination of the virally encoded NLS is essential for the ability of HIV to infect non-dividing cells.

## Synopsis

Human immunodeficiency virus can infect many cells irrespective of whether or not they are dividing, whereas some other retroviruses, such as the murine leukemia virus can only infect cells that are proliferating. This property is important for the ability of HIV to establish infections in critical cell types in infected people. Multiple and redundant signals encoded by HIV have been hypothesized to facilitate migration of viral genomes into the nucleus. However, here the authors eliminated all four described nuclear localizing signals from an HIV genome and show that no combination of these virally encoded signals is essential for the ability of HIV to infect non-dividing cells. They suggest that another step of the virus lifecycle, other than nuclear import, is the rate-limiting step that determines the cell cycle dependence/independence of retroviral infections.

## Introduction

Human immunodeficiency virus and other lentiviruses have the ability to infect non-dividing cells [1–3]. This property allows HIV to integrate into two major types of virus reservoirs in vivo: resting CD4+ T cells and macrophages [4]. However, the ability to productively infect non-dividing cells is not shared by all retroviruses [5]. For example, the gamma retroviruses as exemplified by the murine leukemia virus (MLV) requires mitosis for integration [6,7]. Infection and transduction with foamy retroviruses also depends on cell cycle and requires mitosis [8–10]. An alpharetrovirus, the avian sarcoma virus, appears to be able to integrate viral genomes in non-dividing cells [11,12], but fails to produce virus particles, indicating that it requires mitosis for a later stage of the viral life-cycle [13,14].

After entry into the cytoplasm, retroviruses undergo an uncoating and reverse transcription process that yields a large nucleoprotein complex called the preintegration complex (PIC) [15]. Nuclear entry of viral DNA is an essential step in the retroviral life cycle since viral genomic DNA in the PIC must enter the nucleus to be integrated into host cell chromosomes. The prevailing model to explain the ability of lentiviruses to infect cells independent of the cell cycle is that lentiviruses can target their viral genomes into the nucleus of non-dividing cells via active nuclear transport, while gammaretroviruses that cannot infect non-dividing cells gain an access to the host chromosomes only when the nuclear membrane breaks down at mitosis [6,7]. Thus, it has been hypothesized that the PIC of lentiviruses contain virally encoded nuclear localization signals (NLS), which allow active nuclear transport independent of the cell cycle, whereas the PIC of gammaretroviruses do not contain virally encoded NLS, and thus can not enter the nucleus until mitosis (reviewed in [16]).

Several lentiviral elements that contain a potential NLS and are present in the PIC have been identified including the matrix (MA) [17], integrase (IN) [18], and Viral Protein R (Vpr) [19] proteins and a *cis*-acting element called the central polypurine tract (cPPT) [20]. However, the importance of each of these elements is controversial since subsequent studies have shown that HIV lacking one or several mutations in these NLS elements still retains a significant ability to infect non-dividing cells [16,21–27]. The IN protein is a particularly attractive candidate to mediate nuclear import of HIV genomes since it is part of the PIC through all steps of infection until viral integration, and IN is necessary for nuclear localization and transposition of the yeast elements Ty1 and Ty3 [28–30]. Moreover, HIV IN contains nuclear import activity [18,25,31–34], whereas MLV IN lacks such nuclear import activity [35,36]. However, the role of HIV IN within nuclear import of viral genomes has been difficult to definitively address because mutations or deletions within IN often show pleiotropic effects on virus replication, including assembly, and reverse transcription, in addition to integration (reviewed in [37]). Recent work has suggested that HIV IN itself does not contain an NLS, but rather traffics to the nucleus by virtue of binding the lens epithelium-derived growth factor (LEDGF)/p75 protein [36,38–39].

We recently reported that the capsid protein (CA) is a dominant determinant of retroviral infectivity in non-dividing cells since HIV containing MLV CA lost the ability to infect non-dividing cells, even though it still contains proteins with an NLS [40]. Because HIV CA is not nucleophilic [41,42] and is not stably associated with the HIV PIC [43–48], these data led us to propose that nuclear entry is not the rate-limiting step in the ability of HIV to infect non-dividing cells [40,49]. However, because we were unable to eliminate all of the proposed NLS in HIV, we could not rule out the possibility that the chimeric HIV containing MLV CA masked a pathway usually used by HIV for entry into the nucleus.

Here, we directly tested the involvement of IN within infection of non-dividing cells by constructing an HIV-MLV chimeric virus in which the HIV IN coding sequence was replaced with the IN coding sequence of MLV. Somewhat remarkably, this chimeric virus was infectious at a low level and was able to integrate in an IN-dependent manner. Furthermore, this virus infected non-dividing cells as well as it infected dividing cells.

While individual NLS-containing proteins, in some cases combinations, have been mutated or deleted from HIV in previous studies, it could be argued that the effect of the different NLS are redundant, and therefore HIV still retained some ability to infect non-dividing cells because of the presence of other NLS on other proteins. The ability to generate an HIV-based chimera with MLV IN allowed us to further eliminate all of the other described NLS (MA, Vpr, and the cPPT) from an HIV infectious clone. We report here that this chimeric virus without any of the previously described NLS is still able to infect non-dividing cells. We discuss the possibility that uncoating of the entering viral particle, rather than nuclear import, is the rate-limiting step that determines the cell cycle dependence/independence of retroviral infections.

## Results

### Generation of an Infectious Chimeric HIV-1 with MLV IN That Is Integration-Competent

**Figure 1:**
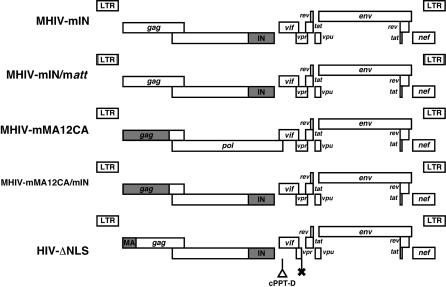
Schematic Representation of the Genomic Organization of Chimeric HIV/MLV Proviruses Portions originated from the HIV genome are shown in white, while those from the MLV genome are in gray. The junction between HIV-1 RT and MLV IN within HIV-mIN was created by direct joining of DNA sequences encoding the C-terminus of HIV RT to the N-terminus of MLV IN. Part of the 3′ end of the HIV-1 IN encoding sequence is retained in the construct of MHIV-mIN to preserve the overlapping Vif sequence and *cis*-acting elements such as cPPT and splice acceptor(s). However, no part of HIV IN should be expressed in the chimeric virus because of the presence of two stop codons following the sequence encoding MLV IN (see Materials and Methods). The molecular clone encoding MHIV-mIN/m*att* has the MLV *att* sites in 5′ U5 and 3′ U3. After reverse transcription, both ends of U5 and U3 will have the MLV *att* sites. MHIV-mMA12CA has been previously described [40]. MHIV-mMA12CA/mIN is similar except it contains MLV IN in addition to the MLV Gag region. A molecular clone of MHIV-mMA12CA/mIN was created by putting the DNA sequence encompassing the MLV IN encoding sequence of the MHIV-mIN with a Vpr mutation into the infectious provirus pMHIV-mMA12CA. HIVΔNLS contains MLV MA instead of HIV MA, MLV IN instead of HIV IN and mutations in the cPPT and in Vpr.

**Figure 2:**
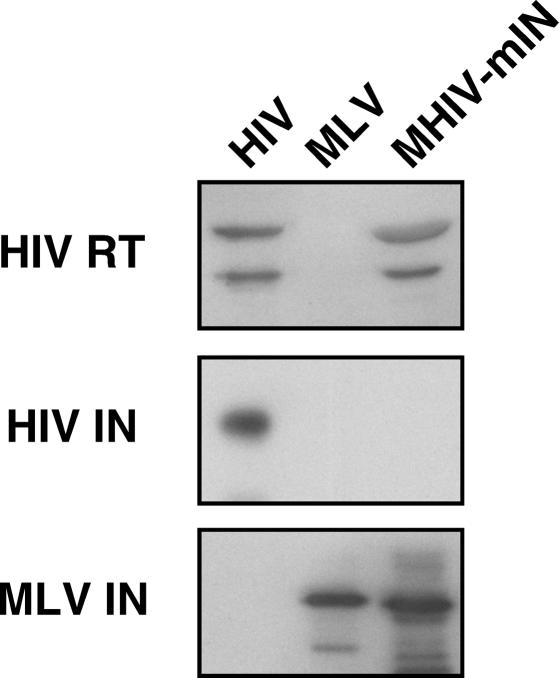
Western Blot Analysis of Purified Virus Particles of MHIV-mIN Together with HIV-1 and MLV Polyprotein processing was tested for HIV-1 RT, HIV-1 IN and MLV IN. Because of low protein production by MHIV-mIN, 10-times more virions were loaded for MHIV-mIN than for HIV-1 and MLV in this experiment.

HIV IN localizes to the nucleus when stably expressed in cells, whereas MLV IN does not [36]. Therefore, to determine if the karyophilic property of IN is essential for the infectivity of HIV in non-dividing cells, HIV-1 IN was replaced with MLV IN within an HIV-based provirus, generating the chimeric clone called MHIV-mIN (which encodes MLV IN instead of HIV IN while the rest of the provirus is HIV) (Figure 1). Transfection of this chimeric provirus showed that it produces virus particles as indicated by the presence of virus-specific proteins in culture supernatants of transfected cells (Figure 2). As expected, MLV IN, and not HIV-1 IN, was detected in virions (Figure 2). The amount of virions made by MHIV-mIN was between 3- to 30-fold lower than that made by wild-type HIV-1 as measured by p24gag ELISA (unpublished data). Nevertheless, processing of reverse transcriptase (RT) and IN appeared normal in virions produced by MHIV-mIN virus (Figure 2).

**Figure 3:**
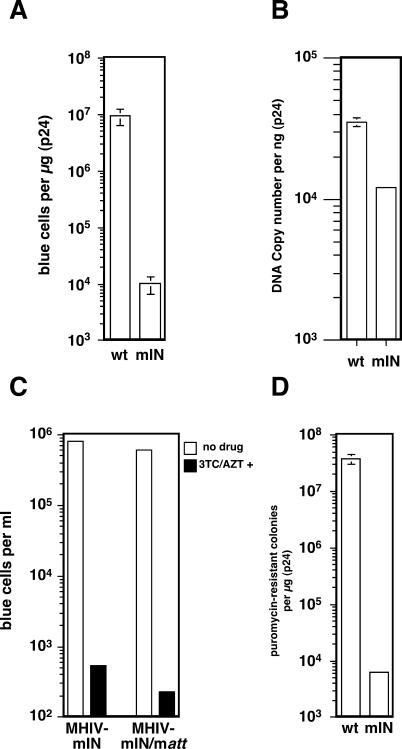
MHIV-mIN Is Infectious (A) Single-cycle infectivity of MHIV-mIN. VSV-G-pseudotyped HIV and MHIV-mIN were made by transfection of 293T cells with plasmid DNA. Infectivity was measured with the MAGI assay by counting β-galactosidase positive cells 2 d post-infection. Virus titers were normalized by the amount of p24 (μg). Infections were performed in triplicate. Mean values are shown here with standard derivation. The background in the assay is about 10 blue cells. (B) Copy numbers of late products of reverse transcription were measured by using real-time quantitative PCR. Viral cDNA numbers were normalized by p24. Infections were performed in triplicate. This is a representative experiment of three independent trials. (C) Infectivity of MHIV-mIN and MHIV-mIN/m*att* was compared in the MAGI assay. VSV-G-pseudotyped viruses were prepared and concentrated at 100-fold by ultracentrifugation. Infections of MAGI cells were also performed in the presence or absence of reverse transcriptase inhibitors (50 μM 3TC and AZT). Formation of blue cells in this assay must result from retrovirus infection since addition of reverse transcription inhibitors (shown as black bars) eliminated most of positive cells. (D) Comparison of infectivity between wild-type HIV-1 and MHIV-mIN as judged by the ability to make puromycin-resistant clones. Infectivity was measured by counting puromycin-resistant colonies 14 d after infection. To facilitate stable transduction of HIV genomes, a mutation was introduced into the *vpr* gene in these proviruses because expression of Vpr would preclude formation of colonies due to its cytotoxicity [69,77]

We tested the infectivity of MHIV-mIN together with wild-type HIV-1 in a single-cycle replication assay [50]. While the titer of MHIV-mIN was about 3-logs lower than that of wild-type HIV-1 when normalized by the amount of p24gag (Figure 3A), it was still well above the background. Real-time PCR data indicated that MHIV-mIN produces 3- to 5-fold less cDNA than wild-type HIV-1 (Figure 3B). Thus, a decrease of reverse transcription of MHIV-mIN alone cannot explain the reduced infectivity of MHIV-mIN.

The integration reaction requires specific recognition by viral IN of short DNA sequences (~10 bp) at both ends of viral DNA, called the attachment (*att*) site. A previous report indicated that replacement of HIV *att* sites with MLV *att* sites at both ends of the long terminal repeat (LTR) reduced viral titer to 0.5% level of the wild-type level [51], while others have found that the *att* sequences other than the conserved CA dinucleotide motif are not very important in vivo [52,53]. To test this, we also made a chimeric clone that contains MLV *att* sequences in both ends of the LTR (Figure 1, called pMHIV-mIN/m*att*), and examined the infectivity of these chimeras (Figure 3C). We could obtain titers of up to 1 × 10^6^ infectious units per ml after concentration of both viruses, but MHIV-mIN/m*att* did not show any significant increase in infectivity when compared with MHIV-mIN (Figure 3C). The infectivity of MHIV-mIN was also sensitive to reverse transcriptase inhibitors (Figure 3C and Figure S1), and thus depends on de novo genomic DNA synthesis. Therefore, we found that there is not a requirement for an MLV-specific *att* site in the context of a chimeric HIV with MLV IN.

IN mutants of HIV that are defective for integration support low levels of infectivity in the multinuclear activation of galactosidase indicate cell (MAGI) assay, probably due to weak expression of the *tat* gene products from unintegrated DNA [54–56]. However stable expression of transduced genes usually requires integration of viral DNA into host chromosome [57,58]. Thus, to genetically test for integration, we made use of a reporter virus system in which the puromycin-resistant gene was put in place of the *nef* gene, and infected cells were selected for puromycin resistance. Compared with HIV, MHIV-mIN exhibits ~4 log decrease of infectivity in the puromycin-based assay (Figure 3D), which is about one log lower than the virus titer difference in MAGI assay (Figure 3A). The difference between the MAGI titer and the puromycin-resistance titer is likely due to expression of Tat from unintegrated DNA [54–56]. Nonetheless, these data show that MHIV-mIN is capable of stable transduction.

**Figure 4:**
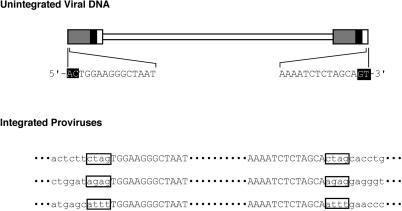
Integration Sites of MHIV-mIN A schematic illustration of the unintegrated viral DNA is shown with the detailed structures of both ends of LTR (top). The two terminal base pairs at each end of the linear DNA precursor, which are removed in the integration process, as shown in highlight. Two other DNA sequences confirmed the removal of dinucleotides from one end of viral DNA (unpublished data). DNA sequences flanking integrated proviral DNA are shown (bottom). Junction sequences between the integrated MHIV-mIN genome and human genomic DNA at each end of the provirus were obtained by nested PCR based on the sequence of integration sites that were mapped with human genome sequences as described in the Materials and Methods. The 4-bp sequence duplications that flank the integrated provirus are shown in boxes.

To more directly address the question of whether or not the MHIV-mIN virus can carry out bona fide IN-mediated integration, we extracted genomic DNA from the puromycin-resistant colonies to amplify and sequence the junction between viral and host sequences. There are two characteristic features of retroviral integration of viral DNA into the host genome. First, two nucleotides are deleted from both ends of viral DNA. Indeed, we observed the deletion of two nucleotides of both ends of all sequenced clones (Figure 4). The second characteristic of IN-mediated integration is that the target sequence of the host DNA is duplicated after the integration event. The size of duplication differs among retroviruses [59]; for example, HIV integration yields 5-bp duplication of the target sequence, whereas MLV integration creates 4-bp duplication. In each case, the length of the duplicated sequence was 4-bp, which is consistent with integration of the HIV chimeric virus mediated by the MLV IN. Taken together these findings demonstrate that MHIV-mIN is competent for all of the early steps of virus replication including integration.

### HIV IN Is Not Essential for Infection of Non-Dividing Cells

**Figure 5:**
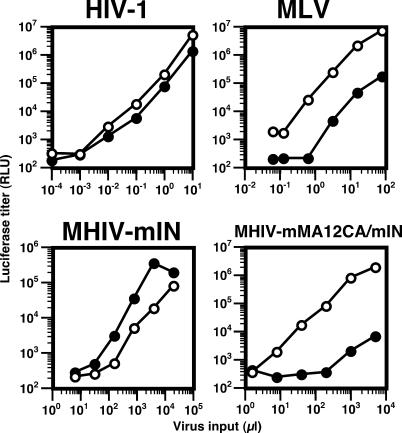
IN Does Not Determine the Infectivity in Non-Dividing Cells Aphidicolin-treated HeLa cells were infected with increasing amount of luciferase-encoding viruses described in Figure 1. Culture supernatants of transfected cells were used as the inoculum. Virus infectivity was judged by measuring luciferase titers of infected cell lysates 2 d after infection. RLU; relative light units. White circles indicate cells without aphidicolin. Filled circles indicate cells with aphidicolin (2 μg per ml). This is a representative experiment that was done at least three times for each virus.

HIV efficiently infects non-dividing cells, whereas MLV infection is restricted in non-dividing cells. To determine if IN plays an essential role in this difference, growth-arrested cells prepared by treatment of HeLa cells with aphidicolin were challenged with the chimeric virus MHIV-mIN along with control viruses, and infectivity was judged by measuring the output of the luciferase gene encoded by reporter virus constructs. As expected from previous studies, wild-type HIV was capable of infecting non-dividing cells as efficiently as dividing cells, while transduction of the luciferase gene by MLV was reduced in non-dividing cells compared with in dividing cells (Figure 5). The phenotype of MHIV-mIN was similar to that with HIV, but not with MLV, in that it was not decreased in non-dividing cells relative to dividing cells (Figure 5). In fact, we saw a slight increase of infectivity by MHIV-mIN on non-dividing cells relative to dividing cells (Figure 5). This increase may be due to expression of the reporter gene from unintegrated DNA in non-dividing cells, as described in the case of infection of non-dividing cells with feline immunodeficiency virus IN mutants [57]. Nonetheless, these results demonstrate that IN is not an essential determinant for the ability of HIV-1 to infect non-dividing cells relative to dividing cells.

We previously showed that replacement of part of the *gag* gene of HIV with that of MLV would convert HIV into a virus that had lost the ability to infect non-dividing cells [40]. Similarly, we found that we can change the phenotype of the MHIV-mIN by replacing the MA and CA proteins of HIV with the MA, p12, and CA proteins of MLV (MHIV-mMA12CA-mIN in Figure 1). Indeed, addition of Gag proteins of MLV into the HIV provirus that already contains MLV IN increased the infectivity in dividing cells, but specifically lost the ability to infect non-dividing cells (Figure 5: Compare MHIV-mMA12CA/mIN with MHIV-mIN). These data demonstrate that Gag, rather than IN, is the dominant determinant for the ability of HIV to infect cells independent of cell cycle progression.

### Normal Levels of Nuclear Import by MHIV-mIN

**Figure 6:**
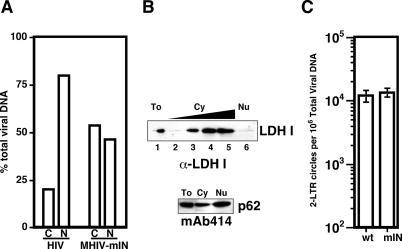
Nuclear Entry of MHIV-mIN (A) Subcellular localization of viral DNA. Infected cells were fractionated to cytoplasmic (C) and nuclear (N) fractions. Viral DNA was extracted and subject to real-time PCR to measure late products of reverse transcription. These data represent one of two independent experiments. Control viruses, with the presence of reverse transcription inhibitors or without VSV-G protein, were also used in the experiments to monitor retrovirus-dependent DNA synthesis, and showed that contamination of plasmid DNA used for transfection is less than 1% of the total DNA. (B) Western blot analysis of total cell lysates (To), cytoplasmic extract (Cy) and nuclear lysates (Nu). Contamination of cytoplasmic extract and the presence of intact cells in nuclear fractions were tested by checking for the presence of a cytoplasmic protein, LDH-I, in each fraction (upper lanes). Five-fold dilutions of the cytoplasmic extract (5-, 25-, and 125-fold dilutions, lanes 4, 3, and 2, respectively) were made to assess the degree of contamination of the nuclear fraction. Presence of proteins was confirmed by antibody against a nuclear pore complex protein (mAb414; lower lane). (C) Nuclear import was monitored by measuring late reverse transcription products and 2-LTR circles. The ratio of total viral DNA and 2-LTR circles was obtained by dividing the copy number of late RT products by the copy number of 2-LTR circle. The parental wild-type strain of HIV-1 (shown here as wt) was compared with the chimeric virus MHIV-mIN (shown as mIN). Control infections with reverse transcription inhibitors (AZT and 3TC: 50 μM each) yielded viral copy numbers that are less than 10% of copy numbers of the samples without reverse transcription inhibitors, indicating that contamination of plasmid DNA used to produce virus stocks does not affect the final results. Two independent experiments gave substantially identical data.

A recent report showed that efficient nuclear entry of HIV can occur independently of mitotic nuclear disassembly in cycling cells [60]. Thus, one interpretation of our results is that elimination of an NLS from HIV would result in lack of infectivity both in dividing and non-dividing cell populations. Indeed, the new chimeric virus created in the present study, MHIV-mIN, infects dividing cells and non-dividing cells with an equal efficiency, but the overall infectivity by MHIV-mIN is severely reduced from that of wild-type HIV-1 (Figure 3A). Thus, to directly determine whether or not MHIV-mIN is restricted at nuclear import of viral DNA, infected cells were separated into cytoplasmic and nuclear fractions and real-time PCR was used to measure late reverse transcription products. The results indicate that there is little apparent difference of viral DNA associated with nuclear fractions between MHIV-mIN and HIV (Figure 6A). Although higher amounts of viral DNA were associated with the nuclear fractions of HIV (~75%) than MHIV-mIN (~50%), this level of difference cannot explain the decrease of infectivity by MHIV-mIN (3-log reduction compared with wild-type HIV). Control experiments using a cytoplasmic protein (LDH I) as a maker for the cytoplasmic fraction indicated that contamination of cytoplasm into the nuclear fraction is less than 1% (Figure 6B. compare the 125-fold dilution of the cytoplasmic fraction in lane 2 with the nuclear fraction in lane 6). These data indicate that nuclear entry of MHIV-mIN is essentially not inhibited and that reduced infectivity of the chimeric virus is due to a post-nuclear entry event (most likely integration). We also examined 2-LTR circles, which are often used as a surrogate marker for nuclear entry. We found that the ratio of 2-LTR circles to total viral DNA in cells infected with MHIV-mIN is roughly equivalent (or even slightly higher) to the ratio in cells infected with parental HIV-1 (Figure 6C). The slight increase in the average number of 2-LTR circles per total viral DNA for MHIV-mIN relative to wild-type virus likely reflects the fact that mutants that integrate inefficiently often accumulate 2-LTR circles [56]. Nonetheless, in sum, these data further support the idea that HIV IN is not essential for the nuclear transport of viral DNA and infectivity in non-dividing cells.

### HIV Lacking All of the Known Types of NLS Still Infects Non-Dividing Cells as Efficiently as Dividing Cells

**Figure 7:**
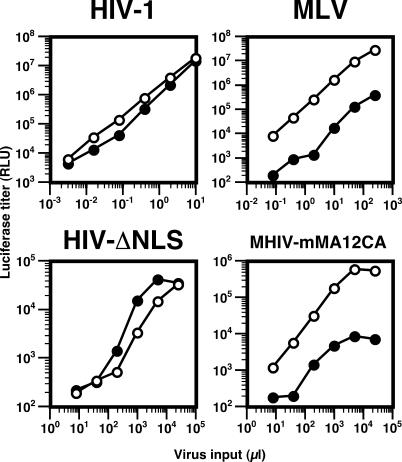
HIV Lacking NLSs Infects Non-Dividing Cells Single-cycle infectivity assay of HIV-ΔNLS together with HIV, MLV, and MHIV-mMA12CA. Both HIV-ΔNLS and MHIV-mMA12CA were concentrated by ultracentrifugation for infections. For details, see the legend to Figure 5. White circles indicate cells without aphidicolin. Filled circles indicate cells with aphidicolin (2 μg per ml). This is a representative experiment that was done at least 3 times for each virus.

As mentioned above, IN is not the sole candidate that potentially encodes a viral NLS. MA, Vpr, and the cPPT have all been described as elements that are important for entry of HIV-1 PIC into the nucleus. To formally address the argument that other described NLSs in HIV as well as the cPPT are redundant for nuclear import with the NLS in HIV IN, a mutant HIV-1 lacking all the NLS candidates was generated. This mutant (HIV-ΔNLS), carrying MLV MA and IN instead of HIV counterparts, lacks a functional *vpr* gene, and has a mutated cPPT (Figure 1). We found that HIV-ΔNLS had reduced infectivity relative to wild-type HIV (Figure 7), but the infectivity of HIV-ΔNLS is sensitive to reverse transcriptase inhibitors (Figure S1), and thus is not an artifact of the virus concentration. Importantly, the infectivity of HIV-ΔNLS is independent of cell cycle conditions (Figure 7). It should be noted that our luciferase system can detect reduction even when the activity is low (See reduction of MLV infectivity in 0.08 μl in Figure 7, for example). The phenotype of HIV-ΔNLS in non-dividing cells is in marked contrast to that of MLV which is dependent on the cell cycle, and in contrast to a previously described chimeric HIV virus containing MLV MA, p12, and CA (MHIV-mMA12CA: Figure 1) [40], which has specifically lost the ability to efficiently infect non-dividing cells (Figure 7). Therefore, these data demonstrate that HIV without any of the previously described NLS elements is fully capable of infecting non-dividing cells as well as it infects dividing cells, and suggest that the virally encoded NLS elements are not rate-limiting for this process.

## Discussion

In the present study, we created a chimeric HIV-1 in which HIV IN is replaced by its counterpart from MLV and demonstrated that HIV can integrate with a heterologous IN protein. Thus, while inefficient, MLV IN can replace HIV IN within the context of an infectious virus. The infectivity of this chimeric virus having MLV IN was not dependent on the presence of the MLV *att* sites at the ends of the LTR, yet did result in duplications of genomic DNA that were consistent with MLV IN. We used this chimeric virus as a template to eliminate all previously described viral NLS elements and found that it was still able to infect non-dividing cells as well as it infected dividing cells.

A popular model for lentiviral infection of non-dividing cells is that the karyophilic activity of an HIV-encoded NLS such as IN is important to bring HIV PIC into the nucleus of non-dividing cells, thereby allowing efficient infection in the absence of mitosis. Our experiments using a chimeric HIV-1 with MLV IN showed that the exchange of IN does not cause any phenotypic change of infectivity that is specific to non-dividing cells. This finding indicates that IN is not an essential determinant that governs the infectious phenotype in non-dividing cells. These results are in agreement with some previous studies that examined individual putative NLS elements within HIV IN and argued against a role of an IN NLS in nuclear import of HIV [16,24,25,61]. Indeed, a recent report by Lu et al demonstrated that mutations in a putative NLS of HIV IN results in class II mutations, which are defective at a postnuclear entry step rather than at nuclear import [61]. In contrast, Ikeda et al claimed that nuclear import of viral DNA is affected by reduced binding of IN to viral cDNA [62]. However, the reduction of nuclear import of such IN mutants (with reduced binding ability to viral cDNA) was at most 40% of the wild-type level as judged by nuclear DNA and that amount of reduction does not seem to explain severely reduced infectivity of their mutants (less than 1% of the wild-type level) [62]. Moreover, other studies have shown that HIV IN localizes to the nucleus by virtue of binding LEDGF/p75 [36,38,39]. However, reduction of LEDGF levels by small interfering RNA (siRNA) affected the nuclear localization of HIV IN, but did not affect the ability of HIV to infect non-dividing cells [36]. Therefore, although we cannot completely rule out the possibility that HIV IN is involved in nuclear migration of viral DNA, we believe that its role is minor.

Although other studies have ruled out a role for individual and some combinations of putative karyophilic viral elements in the HIV PIC, it has not been possible up to now to eliminate all of the identified elements at once in order to test the hypothesis that infection of non-dividing cells is reliant on multiple redundant NLS. However, we were able to create an HIV mutant lacking all of the known NLS-encoding elements, and demonstrated that not only IN, but also none of the other NLS-encoding elements have any effect on the ability of HIV-1 to infect non-dividing cells. Thus, our data are not consistent with a previous suggestion that mutation of single (or double) NLS-encoding elements had little phenotypic change because of redundant NLS-encoding elements that are responsible for nuclear transport of HIV PIC and for infection in non-dividing cells. One possible interpretation of our results is that we have not yet found the most important NLS encoded by HIV. While this is still formally possible, the present results along with our previous results that found that CA is a dominant determinant for retrovirus infectivity in non-dividing cells [40], suggest that these virally encoded karyophilic elements are not the major determinants for the infectivity of HIV in non-dividing cells. Rather, we consider that our data lend support to the alternative hypothesis that nuclear entry is not the rate-limiting step for infection of non-dividing cells. Our hypothesis is also consistent with the findings that the addition of NLS encoding sequences to MLV does not render it infectious to non-dividing cells [35,63].

Instead, we propose that the difference in CA between HIV and MLV affects the progress of uncoating, thereby influencing downstream events such as nuclear import and integration. In this model, uncoating of HIV progresses normally in non-dividing cells and functional PIC enter the nucleus where they integrate viral DNA. In contrast, uncoating of MLV is impaired in non-dividing cells, which results in the failure of subsequent steps of the replication cycle. In this scenario, gammaretroviruses may need mitosis to complete uncoating. In fact, in the case of HIV, CA is dissociated from viral nucleoprotein complexes [43–48], while larger amounts of CA are associated with MLV PICs [15,64–65], suggesting that uncoating of MLV may not be as efficient as that of HIV. An optimal stability of the HIV core appears to be essential for infectivity [66], and complete uncoating may be a prerequisite for nuclear import of PIC. In this hypothesis, the tight association of MLV CA with PIC prevents cellular machinery from interacting with a putative NLS on MLV PICs, thereby retaining PICs within the cytoplasm of interphase cells. On the other hand, the HIV PIC can migrate into the nucleus of interphase cells by using cellular transport machinery. Thus, we are not arguing that nuclear import of the HIV PIC is not essential. Rather, that it is not the rate-limiting step and that cellular rather than viral components of the PIC might play the major role in viral nuclear import after uncoating.

## Materials and Methods

### Nomenclature and construction of proviruses.

Proviruses were named as follows: MHIV-mIN encodes the MLV IN instead of the HIV IN while the rest of the provirus is HIV. MHIV-mIN/m*att* has the MLV IN and MLV *att* sites. MHIV-MA12CA/mIN encodes the MLV IN as well as the MA, p12, and CA proteins of MLV, instead of the HIV MA and CA. HIV and MLV genes were taken from the infectious proviruses pLai [67] and pAMS [68], respectively. The AMS clone encodes a chimeric strain of amphotropic MLV of which 3′ part of the genome was obtained from the amphotropic virus clone 4070A and the 5′ end from MLV-K. The IN region derived from 4070A was cloned into the proviral DNA of HIV-1 in place of part of the HIV IN encoding sequence. The 5′ end of the MLV IN encoding sequence starts at the same position of the original 5′ end of the HIV IN encoding sequence. Therefore, the junction between the HIV RT encoding sequence and the MLV IN encoding sequence is aggaaagtactaATAGAAAACTCAA (HIV sequence is shown in small letters; MLV sequence is shown in capital letters). Part of the 3′ end of the HIV-1 IN encoding sequence was preserved in the construct of MHIV-mIN, since it contains several important *cis*-acting elements such as central polypurine tract (cPPT). The original TAA stop codon for the MLV IN is followed by additional stop codon (TGA) that prevents expression of a possible fusion protein containing MLV Env, since MLV IN encoding sequence also contains the initiation site of MLV Env. Thus, the junction between the MLV IN encoding sequence and HIV sequence is CGTGGAAGCCCTTAATAGTCTgaattc (MLV sequence is shown in capital letters; HIV sequence is shown in small letters; two stop codons are shown in underlined).

A molecular clone of HIV-m*att* was constructed by replacing the *att* sites of HIV with those of MLV in pLai. The 3′ U3 *att* site of HIV-1 (ctggaagggcta) was replaced with the U3 *att* site of MLV (TGAAAGACCCCAA). The 5′ U5 *att* site of HIV-1 (tctctagcag) was replaced with the U5 *att* site of MLV (ggtctttcat). Then, the clone HIV-m*att* was used to create the construct encoding MHIV-mIN/m*att* by swapping the DNA sequence encompassing the MLV IN encoding sequence in the MHIV-mIN. An additional chimeric HIV-1 having both portion of the *gag* gene and IN of MLV was made by replacing the DNA sequence encoding the HIV MA and CA proteins with the DNA sequence of the MLV MA, p12 and CA in the context of the proviral clone MHIV-mIN, resulting in MHIV-mMA12CA/mIN.

An HIV mutant lacking all of the putative NLS encoding genes was made by mutating the cPPT and the *vpr* gene and by replacing the HIV-1 MA and IN with the MLV MA and IN, respectively. The cPPT-D mutation [20] was introduced into pLai by PCR mutagenesis. Construction of a Vpr mutant (pLai-ΔVpr) [69] and MHIV having the MLV MA [40] was reported previously. All of these mutations and replacements were combined together with the *env*-deficient provirus clone pLai-ΔEnv to create the NLS-minus mutant HIV-ΔNLS.

The reporter virus constructs encoding the luciferase gene were made by introducing the luciferase gene from the wild-type Env-minus HIV-1 (pLai-ΔEnv-luc2) [40] into the new HIV-based constructs. The puromycin resistant gene is cloned into the *nef* gene of molecular clones of HIV-1, MHIV-mIN, and MHIV-Mma12CA/mIN. The HIV puromycin resistant constructs were created in the same way as the luciferase constructs as described above. The *vpr* mutant pLai-ΔVpr was used to introduce an insertional mutation in the *vpr* gene of the puromycin virus constructs.

### Western blot analysis.

Western blots were probed with the following antibodies: rabbit anti-HIV-1 RT antibody (through the AIDS Research and Reference Reagent Program, Division of AIDS, NIAID); mouse monoclonal anti-HIV-1 IN antibody (Michael Malim, King's College, London); rabbit anti-MLV IN (Frederick Bushman, University of Pennsylvania, [65]); sheep antibody against LDH I (Cortex Biochem, San Leandro, CA); and mouse monoclonal antibody against nuclear pore complex proteins MAb414 ( Covance, Devner, Pennsylvania) [70]. The membranes were washed for 30 min in wash buffer (PBS containing 0.2% Tween 20) and then incubated with a 1:10,000 dilution of horseradish peroxidase-conjugated antibodies that match with the primary antibody for 60 min at room temperature. The membranes were washed three times for 30 min, and the bound antibody was detected with ECL Plus Western blotting detection reagents (Amersham Biosciences, Little Chalfont, United Kingdom). In some cases, membranes were stripped and reprobed with another primary antibody.

### Infectivity assays.

Vesicular stomatitis virus G protein (VSV-G)-pseudotyped viruses were prepared by transient transfections of 293T cells performed with the FuGene 6 reagent. HIV and MHIV expression plasmids were co-transfected with a VSV-G-expression vector (pL-VSV-G [71]) in addition to pCMV-tat to express the VSV-G for pL-VSV-G. For the production of VSV-G-pseudotyped MLV, the MLV Gag-Pol expression vector (pCS2-mGP) [40] were used along with the murine retrovirus-based vectors [72] encoding the luciferase gene (pLNCluc) [40] as well as the VSV-G construct. To enhance the infectivity, MHIV-mIN and HIV-ΔNLS were concentrated by ultracentrifugation. Briefly, 25–35 ml of culture supernatant of transfected 293T cells were centrifuged at 500 × g for 5 min to remove cell debris and then filtered through a 0.22 μm filter. The supernatant were transferred into ultracentrifuge tubes and centrifuged at 64,000 × g for 90 min within a SW28 rotor (Beckman Instruments, Fullerton, California, United States). The supernatants were carefully removed and 250–350 μl of culture medium was added at 4 °C for 1 hr and freshly used for infection.

Single-cycle infectivity of HIV and MHIV was measured by challenging MAGI cells with serial dilution of virus and staining for β-galactosidase expression as basically described previously [50]. HeLa cells were used for infections with the luciferase reporter virus stocks. Luciferase titer was assayed with the luciferase assay kit (Promega, Madison, Wisconsin, United States) and read on a luminometer. Growth-arrested cells were prepared by treatment with 2 μg per ml of aphidicolin (Sigma, St. Louis, Missouri, United States). Virus binding was enhanced by spinoculation [73] and by addition of 20 μg per ml of DEAE/dextran.

### Quantification of p24gag and viral cDNA.

The p24gag content of the viral supernatants was determined by an enzyme-linked immunosorbent assay (ELISA; Beckman Coulter, Hialeah, Florida, United States.). Late products of reverse transcription and 2-LTR circles of HIV-1 were measured by using real-time PCR based on a previously published protocol [74] as described previously [40].

### Subcellular fractionation.

One day before infection, approximately 5 million HeLa cells were seeded onto four 75 cm^2^ flasks. The cells were challenged either by the VSV-G-pseudotyped HIV-1 or MHIV-mIN. Cells were infected with virus stocks that can synthesize equivalent amount of viral DNA in target cells. Virus stock of MHIV-mIN was concentrated by ultracentrifugation. Both virus stocks were treated with 50 units of Turbo DNase (Ambion, Austin, Texas, United States) per ml at 37 °C for an hour. Infections were performed with the presence of DEAE/dextran (20 μg per ml).

Subcellular fractionations were carried out based on the method described by Yuan et al [75] with minor modifications. One day after infection, cells were washed, tripsinized, and washed once again with phosphate-buffered saline. In order to extract cell lysates and DNA from intact cells, 20% of the infected cells were kept for further experiments. All the manipulations after this step were carried out at 4 °C. The remaining 80% cells were resuspended in 3 volumes of hypotonic buffer (10 mM HEPES, [pH 7.9]; 1.5 mM MgCl_2_; 10 mM KCl; 2 mM dithiothereitol ; 20 μg of aprotinin per ml). Resuspended cells were centrifuged at 2,300 × g for 5 min. The cell pellet was resuspended in 3 volumes of hypotonic buffer and kept on ice for 10 min. The cells were homogenized with 30 strokes in a Dounce homogenizer. Nuclei and cell debris were pelleted by centrifugation at 3,300 × g for 15 min. The supernatant of this centrifugation was directly used to extract viral DNA or additionally spun down at 13,400 × g for 20 min. The nuclear pellet were washed with 3 volumes of hypotonic buffer containing 0.005% digitonin once and then washed with hypotonic buffer twice. DNA was extracted from half of each fraction using the QIAamp DNA Mini Kit (Qiagen, Valencia, California, United States), and the other half was used for Western blotting to assess the integrity of the fractionation procedure using antibodies to a cytoplasmic protein or a nuclear pore antigen.

To assess the integrity of the fractionation procedure, we examined the contamination of cytoplasmic fraction into nuclear fraction by monitoring the presence of LDH I. Intact cells (10% of the infected cells) and nuclei (half of the purified nuclei) were first resuspended with 50 μl and 100 μl of NTE buffer (10 mM Tris-HCl, [pH 8.0]; 1 mM EDTA; 50 mM NaCl; 2 mM DTT; 20 μg of aprotinin per ml), respectively. After incubation on ice for 5 min, equivalent amount of NP40-doc buffer (1% NP40; 0.2% sodium deoxycholate; 0.12 M NaCl; 20 mM Tris-HCl, [pH 8.0]) were added to the samples and kept on ice for 10 min. The samples were mixed by vortex and spun down at 9,300 × g for 5 min. Twenty μg of protein samples was used in SDS-PAGE and western blotting analysis.

The most serious problem for our experiments was potential contamination of cytoplasmic viral DNA into purified nuclei. Viral DNA is present in a nucleoprotein complex or a free-DNA form, and those viral DNA may behave differently than cytoplasmic proteins such as LDH I in the process of fractionation. To address this possibility, we used a control to determine if quantity of contamination of viral DNA from the cytoplasmic fraction into the nuclear fraction during the washing steps. To this end, we mixed cytoplasmic extracts of infected cells with nuclei of uninfected cells. In these experiments, we used MLV instead of HIV because of the ease of manipulation. Virus stocks of MLV were prepared by harvesting culture supernatant of ecotropic MLV-producing NIH/3T3 cells. Cytoplasmic extracts of acutely infected cells and nuclei of uninfected NIH/3T3 cells were prepared, mixed on ice, and washed as described above. Nuclear-associated DNA was extracted and subject to real-time PCR to measure the copy number of late reverse transcription products as described above. We found that there was less than 1% introduction of cytoplasmic viral DNA into the nuclear fraction during the washing steps (unpublished data).

### Sequencing of junctions between host DNA and integrated viral DNA.

Junction sequences between host DNA and viral DNA were determined by using an inverse PCR strategy as described before [76]. HeLa cells were infected with VSV-G-pseudotyped MHIV-mIN-ΔVpr-Puro. Puromycin-resistant cell clones (~130 colonies) were selected for 2–3 weeks with the presence of puromycin (0.7 μg per ml) and used to extract genomic DNA. Genomic DNA (2 μg) from infected cells was digested with 20 U of PstI at 37 °C for 12 h. After heat inactivation at 65 °C for 40 min, 200 ng of digested DNA were taken out for ligation reaction. The ligation reaction was carried out at 16 °C for 12 h. Ligase was then heat inactivated at 65 °C for 15 min. The region of the junction between cellular DNA and the 5′ end of the integrated proviral DNA was amplified by nested inverse PCR. The first PCR primers were U3RRG2, 5′-GGCAAGCTTTATTGAGGC-3′and Gag716, 5′-GGTCAGCCAAAATTACCCTATAGTG-3′. The second PCR primers were MH536, 5′-TCCACAGATCAAGGATATCTTGTC-3′, and Gag934, 5′-TGTTAAAAGAGACCATCAATGAGGAAG-3′. The PCR was carried out in 50 μl solution, which contains 1 × PCR buffer, dNTPs (0.2 mM), primers (1 μM), 10 units of Taq polymerase (Roche, Basel, Switzerland), and 200 ng of ligated DNA. PCR products were purified and used for cloning by using pGEM-T Vector System (Promega). Positive clones were sequenced by using the T7 primer.

Junction sequences between 3′ ends of viral DNA and host DNA were determined for three of the clones by nested PCR with 5′sense primers matching with 3′ LTR of proviral DNA and with 3′ anti-sense primers matching with host DNA downstream of viral DNA. The information obtained from 5′ junction sequences between host DNA and viral DNA allowed us to map integration sites of these three clones in the human genome sequence deposited in GenBank. Based on this information, 3′ primers were designed. Amplified products were cloned into T-vector and sequenced.

## Supporting Information

Figure S1 Single-Cycle Infectivity Assay of MHIV-mIN and HIV-ΔNLS with Reverse Transcriptase Inhibitors Infections with viruses that encode the luciferase gene in place of *nef* were performed with the presence (shown in black) or absence (shown in gray) of a reverse transcriptase inhibitor (RTI) (AZT and 3TC: 50 μM each). For details, see the legend to Figure 5. In both cases, the luciferase activity is decreased by RTI which indicates that expression of luciferase relies on de novo RT activity. Also, the presence of aphidicolin does not change the dependence on de novo reverse transcriptase activity for luciferase activity. This is a representative experiment done with two different virus stocks with virtually identical results.
(15 MB TIF)

## Abbreviations

CA: capsid protein
cPPT: central polypurine tract
IN: integrase
MA: matrix
MLV: murine leukemia virus
NLS: nuclear localization signal
PIC: preintegration complex
RT: reverse transcriptase
RTI: reverse transcriptase inhibitor
Vpr: Viral Protein R
VSV-G: Vesicular stomatitis virus G protein
